# Adenovirus-mediated interleukin-12 gene transfer combined with *cytosine deaminase *followed by 5-fluorocytosine treatment exerts potent antitumor activity in Renca tumor-bearing mice

**DOI:** 10.1186/1471-2407-5-51

**Published:** 2005-05-24

**Authors:** Kyung-Sun Hwang, Won-Kyung Cho, Jinsang Yoo, Hwan-Jung Yun, Samyong Kim, Dong-Soo Im

**Affiliations:** 1 Laboratory of Gene Therapy and Virology, Korea Research Institute of Bioscience and Biotechnology, Yusong, Daejeon, Republic of Korea; 2 Department of Internal Medicine, College of Medicine, Chungnam National University, Daejeon, Republic of Korea

## Abstract

**Background:**

Therapeutic gene transfer affords a clinically feasible and safe approach to cancer treatment but a more effective modality is needed to improve clinical outcomes. Combined transfer of therapeutic genes with different modes of actions may be a means to this end. Interleukin-12 (IL-12), a heterodimeric immunoregulatory cytokine composed of covalently linked p35 and p40 subunits, has antitumor activity in animal models. The enzyme/prodrug strategy using cytosine deaminase (CD) and 5-fluorocytosine (5-FC) has been used for cancer gene therapy. We have evaluated the antitumor effect of combining IL-12 with CD gene transfer in mice bearing renal cell carcinoma (Renca) tumors.

**Methods:**

Adenoviral vectors were constructed encoding one or both subunits of murine IL-12 (Ad.p35, Ad.p40 and Ad.IL-12) or cytosine deaminase (Ad.CD). The functionality of the IL-12 or CD gene products expressed from these vectors was validated by splenic interferon (IFN)-γ production or viability assays in cultured cells. Ad.p35 plus Ad.p40, or Ad.IL-12, with or without Ad.CD, were administered (single-dose) intratumorally to Renca tumor-bearing mice. The animals injected with Ad.CD also received 5-FC intraperitoneally. The antitumor effects were then evaluated by measuring tumor regression, mean animal survival time, splenic natural killer (NK) cell activity and IFN-γ production.

**Results:**

The inhibition of tumor growth in mice treated with Ad.p35 plus Ad.p40 and Ad.CD, followed by injection of 5-FC, was significantly greater than that in mice treated with Ad.CD/5-FC, a mixture of Ad.p35 plus Ad.p40, or Ad.GFP (control). The combined gene transfer increased splenic NK cell activity and IFN-γ production by splenocytes. Ad.CD/5-FC treatment significantly increased the antitumor effect of Ad.IL-12 in terms of tumor growth inhibition and mean animal survival time.

**Conclusion:**

The results suggest that adenovirus-mediated IL-12 gene transfer combined with Ad.CD followed by 5-FC treatment may be useful for treating cancers.

## Background

Gene therapy for treating cancers has been intensively investigated. Therapeutic gene transfer using viral or nonviral vectors has been shown to be clinically feasible and safe. However, the therapeutic outcome appears not to be high as expected, presumably because the in vivo gene delivery efficiency is low. In this sense, a more effective modality is needed to improve the therapeutic benefit. Combined transfer of genes with different mechanisms of action may potentate the therapeutic outcome. Indeed, combination cancer gene therapy using cytokine and suicide genes has been shown to be more effective than single gene transfer in animal models [1-9].

Interleukin (IL)-12 is a heterodimeric cytokine composed of 35- and 40-kDa subunits, which are covalently linked by a disulfide bond [10,11]. IL-12 stimulates interferon-γ (IFN-γ) production by natural killer (NK) cells, enhances the cytolytic activities of NK cells and cytotoxic T lymphocytes (CTLs), stimulates the differentiation of Th1 cells and inhibits angiogenesis [12-14]. These biological activities have provided a rationale for the use of IL-12 in cancer immunotherapy. In fact, recombinant IL-12 (rIL-12) exhibits significant antitumor activity in various animal models [12-14]. Subsequent phase I and II studies on various cancer types using intravenous or subcutaneous administration of human rIL-12 [15,16] showed some beneficial responses, but there were also adverse effects, which interrupted the clinical trials [17,18]. Recently, it has been reported that intratumoral administration of rIL-12 shows dose-limiting toxicity and results in measurable locoregional immunological responses in head and neck squamous cell carcinoma patients [19].

Since intratumoral IL-12 gene transfer could minimize the adverse effects of systemic rIL-12 administration [17], the effectiveness against tumors of cell vaccines expressing IL-12, or viral or nonviral vectors encoding IL-12, has been evaluated in various animal models [18,20-23]. The results indicate that, compared to IL-2, IL-4, interferons, and granulocyte-macrophage colony-stimulating factor, IL-12 is one of the most effective immunomodulators for cancer treatment [13,18,20,21,23]. Pilot clinical trials have been conducted in patients with advanced primary and metastatic liver cancer on the basis of these results [24]. Intratumorally administered adenoviral vectors expressing IL-12 had low efficacy but no cumulative toxicity [24].

Suicide gene therapy deploys genes such as cytosine deaminase (CD), herpes simplex or varicella zoster virus thymidine kinase (TK), or nitroreductase, all of which convert nontoxic prodrugs into cytotoxic agents [25-27]. Systemically administered prodrugs are converted to the active agents only in cells expressing these suicide genes, so the therapeutic effect is maximized while systemic toxicity is reduced. CD, which normally catalyzes the deamination of cytosine to uracil during RNA biosynthesis, is found in many bacteria and fungi but not in mammalian cells [28-30]. It also deaminates 5-fluorocytosine (5-FC) to 5-fluorouracil (5-FU), which is highly toxic to cells. 5-FU has been used clinically since the late 1950s to treat colorectal cancer [31]. A cellular metabolite of 5-FU, 5-fluorouridine triphosphate, is incorporated into RNA in place of UTP and consequently the multiple functions of RNA are impaired. Another metabolite of 5-FU, 5-fluorodeoxyuridine monophosphate, blocks thymidylate synthase activity and thus inhibits DNA synthesis.

No currently available gene delivery system can deliver therapeutic genes including suicide genes to all the target cells *in vivo*. However, tumor cells expressing a suicide gene can kill neighboring tumor cells that do not express this gene by the by-stander effect, which could partly overcome the gene delivery problem [32-35]. A number of suicide gene therapies have been shown to be effective against various cancer cell lines in culture and in animal models. Subsequent phase I, II or III studies using CD or TK genes were performed in colorectal cancer patients with liver metastases and in brain tumor patients [36-47]. The antitumor activities appeared less than expected [46], but the results showed that suicide gene therapy is safe to apply in the oncology clinic [36-47].

A combination of IL-2 and TK genes exerted a more potent therapeutic outcome in a metastatic carcinoma model, and induced antitumor immunity [1]. Since then, combination gene therapies using various cytokine and suicide genes have been intensively investigated [2-9]. In the present study, we investigated the effectiveness of combined transfer of IL-12 and CD genes against renal cell carcinoma (Renca)-tumors in mice.

## Methods

### Cells and mice

HeLa, 293, Renca (a cell line spontaneously derived from BALB/c renal cell adenocarcinoma), and NK cell-sensitive YAC-1 cells were cultured in DMEM or RPMI-1640 medium (Gibco-BRL) supplemented with 10% fetal bovine serum (FBS) and penicillin/streptomycin. Cultures were maintained in a 5% CO_2 _atmosphere at 37°C.

Male or female wild-type BALB/c mice, 7–8 weeks of age, were purchased from the animal breeding laboratory at the Korea Research Institute of Bioscience and Biotechnology. They were kept (five mice per cage) in isolation under specific pathogen-free conditions, exposed to 12-h light/12-h dark cycles, and provided with standard feed and water *ad libitum*.

### Construction of replication-defective adenoviral vectors

cDNAs for *Esherichia coli *cytosine deaminase, p35 and/or p40 murine IL-12 subunits [48] or green fluorescent protein (GFP) were inserted by a standard cloning procedure into pX.dCMV.pA, an adeno-transfer plasmid harboring the immediate early gene promoter from human cytomegalovirus [49-51]. The shuttle plasmids were cotransfected into 293 cells together with pJM17, which harbors the genomic DNA of adenovirus 5 (Microbix, Canada), by a standard calcium phosphate method. Primary plaques were isolated and screened by PCR to ensure the presence of the inserts. Positive clones were plaque-purified twice and a single plaque was chosen for further amplification in 293 cells. Recombinant adenoviruses were purified by cesium chloride gradient centrifugation as previously described [49-51], dialyzed to remove the cesium chloride and frozen at -70°C.The viral titer was determined by a 293 plaque assay and expressed as plaque-forming units (pfu).

### Cell viability assay

HeLa cells were plated on 60-mm dishes at 5 × 10^5 ^cells/plate and mock-transduced, or transduced with Ad.CD, for 2 h at a multiplicity of infection (MOI) of 50 in serum-free DMEM. Cultures were continued for a further 12 h. Transduced and non-transduced cells were then mixed in different ratios and incubated in 24-well dishes for 24 h. The culture media were replaced with fresh media containing various concentrations of 5-FC, and incubation was continued for 4 days. Cell viability was assessed by the trypan-blue exclusion assay.

### Western blotting

HeLa cells were plated on 60-mm dishes at 5 × 10^5 ^cells/dish and transduced with recombinant adenoviruses in serum-free DMEM for 2 h. The culture media from mock- or virus-transduced cells were retained for measurement of released IL-12 at 48 h postinfection, and the cells were harvested in a lysis buffer (10 mM Tris-HCl, pH 7.5, 10 mM NaCl, 1.5 mM MgCl_2_, 10 mM β-mercaptoethanol). Cell extracts were prepared by three cycles of freezing (-70°C) and thawing and precleared by centrifugation. Fifty micrograms of precleared cell lysates were subjected to SDS-10% polyacrylamide gel electrophoresis (PAGE). The separated proteins were transferred to PVDF membranes, which were blocked with 3% bovine serum albumin (BSA) in phosphate-buffered saline (PBS) and incubated with rat anti-mouse IL-12 antibody (PharMingen), then with horseradish peroxidase-conjugated rabbit anti-rat lgG antibody (Sigma). Proteins were detected according to Amersham's ECL protocol. The culture supernatants were incubated with anti-IL-12 antibody. A suspension of protein G-Sepharose beads (100 μl) was added and the incubation mixtures were further incubated at 4°C for 12 h. The immunoprecipitates were washed three times with 40 mM N-2-hydroxyethylpiperazine-N'-2-ethanesulfonic acid, pH 7.4, 100 mM KCl, 0.1% NP-40, 0.1 mM DTT, and once with PBS, then immunoblotted with the rat anti-mouse IL-12 antibody described above.

### Animal model and treatment

Tumors were generated on the flanks of BALB/c mice by subcutaneous (s.c.) injection of 1.5 × 10^5 ^of Renca cells in 0.1 ml of PBS. After visible tumors had developed for 7–9 days after inoculation, the mice were injected once intratumorally with recombinant adenoviruses at 1 × 10^9 ^pfu in 10 mM Tris-HCl, pH 7.4, 1 mM MgCl_2_, 10% glycerol. Animals undergoing 5-fluorocytosine therapy received intraperitoneal administration of 5-FC (400 mg/kg) daily for 10 days. Tumors were measured prior to virus injection and subsequently at intervals of 3 days. Linear calipers were used to measure the longest diameter (a), width (b), and depth (c). The tumor size was calculated by the formula: (a × b × c/2).

### ELISA for IFN-γ

ELISAs were performed using essentially the reagents and procedures described in the Endogen (USA) product literature. Flat-bottomed 96-well microtiter plates (Nunc Maxisorp) were coated with rat anti-IFN-γ antibodies (1 μg/ml) at 4°C overnight and blocked with 4% BSA in PBS for 2 h at 37°C. Renca cells were treated with mitomycin C at 50 μg/ml for 20 min at 37°C. Splenocytes (2 × 10^6 ^cells/ml) obtained from the tumor-bearing mice 21 days after virus treatment were stimulated with the inactivated Renca cells in U-bottomed 96-well plates at an effector-to-stimulator ratio of 10:1. Supernatants (50 μl) from 24 h and 48 h cultures were added to the ELISA plate wells and incubated for 1 h. After several washing steps, rat anti-mouse IFN-γ antibody conjugated with biotin was added. Color was developed using avidin-conjugated horseradish peroxidase and OPD substrate (Sigma). The reaction was terminated by the addition of 2N H_2_SO_4_. The absorbance was measured at 490 nm with an ELISA plate reader. The amounts of IFN-γ were quantified by interpolation of a standard curve generated using known amounts of standard mouse rIFN-γ (Endogen). The biological activity of the IL-12 produced from the recombinant adenoviruses was assessed on the basis of its ability to induce IFN-γ secretion from murine splenocytes. Murine splenocytes (1.5 × 10^6 ^cells/well) were washed with cold medium and incubated with the culture supernatants. IFN-γ in the incubation media was then determined by an ELISA, as described above.

### Cytotoxicity assay of NK cells

The cytolytic activity of NK cells was determined by a standard 4-h ^51^Cr-release assay. The spleens from dead mice obtained 21 days after tumor inoculation were removed aseptically and single-cell suspensions, prepared by passing the spleens through a metal mesh, were suspended in 5 ml of 0.83% ammonium chloride to lyse the red blood cells. The splenocytes were then used as effector cells to assay NK cell activity. YAC-1 cells (2 × 10^6^) in 0.5 ml of RPMI-1640 with 20% FBS were labeled with 50 μCi of Na^51^CrO_4 _(Amersham) for 90 min. The labeled cells were washed three times with serum-free DMEM and mixed (1 × 10^4 ^per well) with the effector cells in a U-bottomed 96-well plate for 4 h at 37°C at three different effector-to-target ratios (100:1, 33:1, and 11:1), each in triplicate. The mixtures of target and effector cells were incubated for 4 h at 37°C. The percentage of specific ^51^Cr release was calculated as [(cpm experimental release - cpm spontaneous release/cpm maximum release - cpm spontaneous release)] × 100. The maximum ^51^Cr release was determined from the supernatant when labeled cells were lysed by adding 0.1 ml of 3% SDS. Spontaneous release was determined from the supernatants after labeled cells were incubated in 0.1 ml of medium without effector cells. The standard deviations for each triplicate sample and for spontaneous release were less than 10% and 20%, respectively.

### Statistics

Statistical analysis was carried out using the Student's *t *test (unipolar, paired) and the chi-square test (two-tailed). The values were considered statistically significant when the *P *value was less than 0.05.

## Results and discussion

### Recombinant adenoviruses encoding the p35 and/or p40 subunits of murine IL-12 express bioactive IL-12

**Figure 1:**
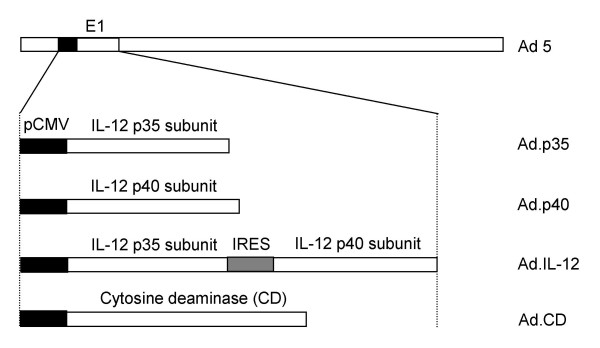
Schematic illustration of replication-defective adenoviral vectors constructed in this study. The E1 region of adenovirus type 5 (Ad 5) was replaced by the indicated genes under the transcriptional control of the immediate early gene promoter from human cytomegalovirus (pCMV). IRES; internal ribosomal entry sequence.

**Figure 2:**
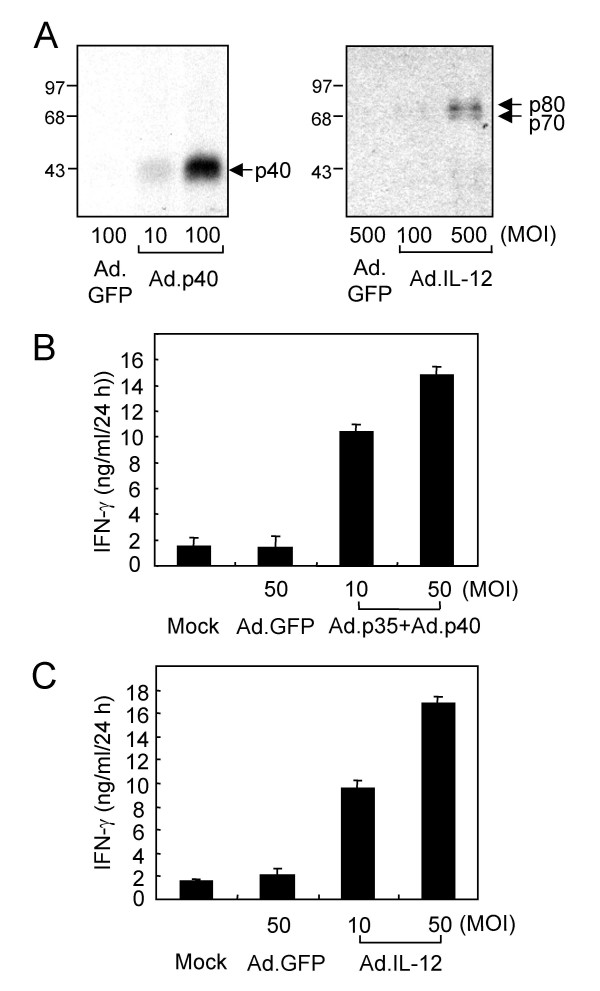
The recombinant adenoviruses encoding murine p35 and/or p40 subunits express biologically active IL-12. (A) HeLa cells were transduced with Ad.GFP, Ad.p40, or Ad.IL-12 at the indicated MOIs. Cells or culture media were harvested 48 h after transduction. Cleared lysates of cells transduced with Ad.GFP or Ad.p40 were immunoblotted with anti-IL-12 antibody (left panel). The culture media from HeLa cells transduced with Ad.IL-12 or Ad.GFP were immunoprecipitated with anti-IL-12 antibody. The immunoprecipitates were dissolved in SDS sample buffer without reducing agent and subjected to SDS-PAGE, followed by immunoblotting with anti-IL-12 antibody (right panel). Molecular size markers in kilodaltons are shown at the left. The positions of p40, p35–p40 heterodimer (p70) and p40 homodimer (p80) are indicated by arrows. (B and C) HeLa cells were mock-transduced or transduced with either a 1:1 (pfu ratio) mixture of Ad.p35 and Ad.p40, or Ad.IL-12, or Ad.GFP, at the indicated MOI. Splenocytes from naïve mice were incubated with the culture supernatants for 24 h. ELISA was performed to determine the IFN-γ levels after incubation. Data are means ± SD of two independent experiments, each performed in duplicate.

We constructed replication-defective adenoviruses containing the p35 and/or p40 subunits of murine IL-12 under the transcriptional control of the immediate early gene promoter of human cytomegalovirus (Figure 1). Ad.IL-12 contains murine cDNAs of both IL-12 subunits, which are linked by an internal ribosome entry site sequence from encephalomyocarditis virus. To test whether Ad.p40 and Ad.IL-12 express the p40 subunit and the p35-p40 heterodimer respectively, we transduced HeLa cells with the two constructs at the indicated MOI. Replication-defective adenovirus encoding GFP (Ad.GFP) was used as a control. Lysates of cells transduced with Ad.p40 or Ad.GFP were resolved by SDS-PAGE followed by immunoblotting with anti-IL-12 antibody, which reacts specifically with the p40 subunit of mouse IL-12 and with free p40 (Figure 2A, left panel). Ad.p40 was found to express p40. The culture media from cells transduced with Ad.IL-12 or Ad.GFP were immunoprecipitated with anti-IL-12 antibody. The immuno-complexes were resolved by SDS-PAGE under nonreducing conditions and immunoblotted with anti-IL-12 antibody. The two protein bands detected might be a p40 homodimer (p80) and a p35-p40 heterodimer (p70) (Figure 2A, right panel, indicated by arrows). Under nonreducing conditions, Ad.IL-12 produced a doublet of 79/75 kDa that was sensitive to a reducing agent [52]. Ad.p40 produces a band migrating with an apparent molecular mass of approximately 90 kDa, which on a reducing gel reveals a band consistent with the p40 monomer [53]. Thus, our result appeared to be consistent with previous findings. We could not assess p35 expression because no antibody specific for this subunit was available.

We next examined whether cells transduced with adenoviruses encoding the p35 and/or p40 subunits release bioactive IL-12. HeLa cells were mock-transduced, or transduced with a 1:1 (pfu ratio) mixture of Ad.p35 and Ad.p40, Ad.IL-12, or Ad.GFP at the indicated MOI, and the culture supernatants were collected after 24 h incubation. Splenocytes from naïve mice were incubated with equivalent volumes of the culture supernatants, and the IFN-γ contents of the medium were measured using ELISA (Figure 2B and 2C). The culture supernatants from cells transduced with a mixture of Ad.p35 and Ad.p40 or Ad.IL-12 exhibited much higher IFN-γ inducing-activity than the controls. These results suggest that cells transduced with Ad.p35 plus Ad.p40 or Ad.IL-12 release bioactive IL-12.

### Recombinant adenovirus encoding cytosine deaminase (Ad.CD) expresses catalytically active cytosine deaminase

**Figure 3:**
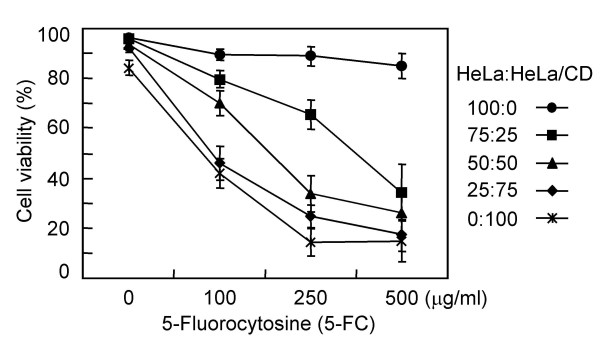
Ad.CD virus expresses functional *cytosine deaminase*. HeLa cells were transduced with Ad.CD at an MOI of 50 and incubated for 12 h. These cells (HeLa/CD) were then mixed with naïve HeLa cells at the indicated ratios. The HeLa/CD, HeLa, or mixed cells were incubated on 24-well dishes for 24 h, then treated with 5-FC at the indicated concentrations for 4 days. Cell viability was assessed by the trypan-blue exclusion assay. Data are means ± SD of two independent experiments, each performed in duplicate.

We constructed a replication-defective adenovirus encoding cytosine deaminase (Figure 1). To examine whether the Ad.CD virus expresses cytosine deaminase capable of converting 5-FC to cytotoxic 5-FU, we transduced HeLa cells with Ad.CD at an MOI of 50. Naïve HeLa or HeLa/CD cells, or mixtures of the two at the indicated ratios (Figure 3), were incubated in culture media without or with the indicated concentrations of 5-FC for 4 days, and cell viability was assessed by the trypan-blue exclusion assay (Figure 3). In the presence of 5-FC, the viability of the HeLa/CD cells decreased markedly but the naïve HeLa cells were unaffected, suggesting that cytosine deaminase was expressed in the virus-transduced cells and metabolized 5-FC to 5-FU. The viability of the mixtures of naïve and HeLa/CD cells appeared to decrease more than the proportion of naïve cells in the mixtures, particularly with 500 μg/ml 5-FC. Thus, the Ad.CD virus appeared to exert a bystander effect, though not a drastic one. The results suggest that Ad.CD expresses catalytically active cytosine deaminase.

### Transfer of the IL-12 and *cytosine deaminase *genes in combination, followed by 5-FC treatment, exerts potent antitumor activity in Renca-tumor bearing mice

**Figure 4:**
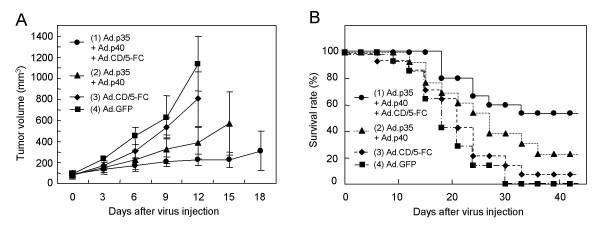
The antitumor activity effected by IL-12 gene transfer increases *in vivo *when combined with *cytosine deaminase*/5-FC. (A) Renca tumors were established in BALB/c mice by s.c. inoculation of 1.5 × 10^5 ^cells. When the tumors reached 5–6 mm diameter, the animals were randomly divided into four treatment groups (n = 15 per group) and injected once intratumorally with (1) Ad.p35 (2.5 × 10^8 ^pfu) plus Ad.p40 (2.5 × 10^8 ^pfu) plus Ad.CD (5 × 10^8 ^pfu), or (2) Ad.p35 (5 × 10^8 ^pfu) and Ad.p40 (5 × 10^8 ^pfu), or (3) Ad.CD (1 × 10^9 ^pfu), or (4) Ad.GFP (1 × 10^9 ^pfu). Mice treated with Ad.CD were given 5-FC (400 mg/kg) intraperitoneally daily for 10 days. Graphic representations of mean tumor growth rates are shown. Bars indicate SD. (B) Mouse survival until day 42 was recorded. Mice were sacrificed and considered as death when tumor size exceeded 1.5 cm in long and short axes. The mean survival times (± SD) were 33.2 ± 10.4 days for (1) group, 27.2 ± 10.9 days for (2) group, 21.9 ± 9.1 days for (3) group, and 19.7 ± 7.1 days for (4) group. The difference between (1) and (2) groups was statistically significant (*p *< 0.0018) by Student's *t *test. The treatment outcome is summarized in Table 1. One animal in each of the groups treated with Ad.CD and Ad.GFP succumbed to toxicity, and two mice in the group treated with Ad.p35 plus Ad.p40 were excluded owing to peritoneal metastasis.

Renca-tumor bearing mice were established as described in the Methods section, randomly divided into 4 groups, and injected once intratumorally with Ad.GFP, Ad.CD/5-FC, Ad.p35 plus Ad.p40, or Ad.p35 plus Ad.p40 plus Ad.CD. Mice receiving Ad.CD were treated with 5-FC. Tumor growth was measured at the indicated times (Figure 4A). Mouse survival until day 42 was recorded (Figure 4B). The treatment outcomes are summarized in Table 1. Inhibition of tumor growth was most marked in mice treated with Ad.p35 plus Ad.p40 plus Ad.CD/5-FC (*p *< 0.001 for Ad.p35 plus Ad.p40 plus Ad.CD/5-FC vs Ad.GFP and *p *< 0.0053 for Ad.p35 plus Ad.p40 plus Ad.CD/5-FC vs Ad.CD/5-FC). The mean survival times were for 33.2 ± 10.4 days for Ad.p35 plus Ad.p40 plus Ad.CD/5-FC group and 27.2 ± 10.9 days for Ad.p35 plus Ad.p40 group; the difference is statistically significant (*p *< 0.0018). Among the 15 mice treated with Ad.p35 plus Ad.p40 plus Ad.CD/5-FC, four became tumor-free and four exhibited partial responses (Table 1). Among the 15 mice treated with Ad.p35 plus Ad.p40, one became tumor-free and two showed partial responses (*p *< 0.0464 for comparison of the two treatments). Ad.p35 plus Ad.p40 treatment appeared to be more effective than Ad.CD/5-FC, which had a modest antitumor effect compared to the control (Ad.GFP).

### IL-12 and *cytosine deaminase *genes in combination, followed by 5-FC treatment, enhance the cytolytic activity of NK cells and increase IFN-γ production

**Figure 5:**
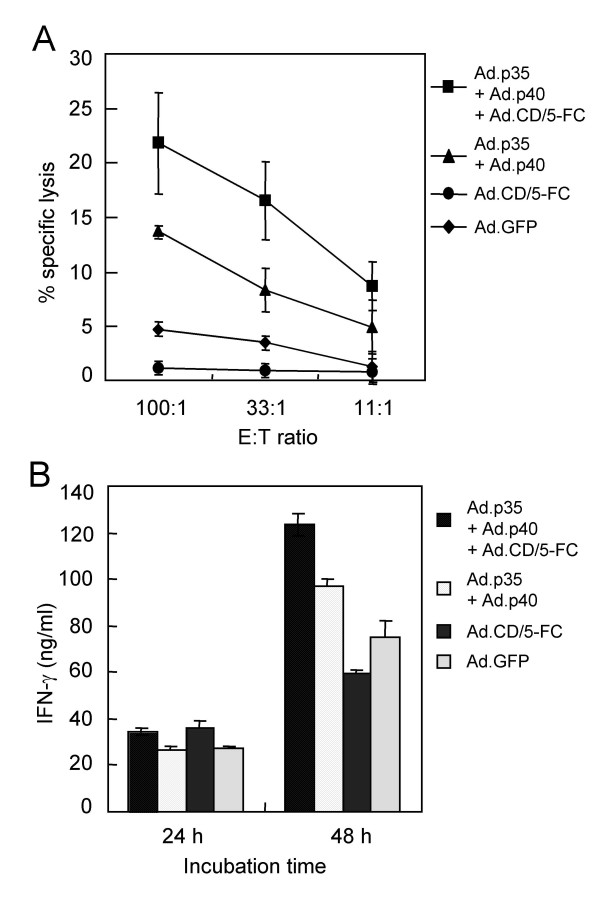
IL-12 gene transfer combined with Ad.CD/5-FC increases splenic NK cell activity and IFN-γ production. (A) Cytotoxicity of NK cells in Renca tumor-bearing mice treated with various adenoviral vectors (see Figure 4 legend). Mice in which tumor growth was completely or partially inhibited were chosen from the treatment groups on day 21 after tumor cell inoculation. Splenocytes were isolated from the treated mice (n = 3 per group) and used as effector cells. A standard 4-h ^51^Cr release assay was performed using YAC-1 cells labeled with ^51^Cr as target cells. The percentage specific lysis was determined. Data are means ± SD. (B) Mouse splenocytes (n = 3 per group) were isolated from the treatment groups on day 21 as described above, and incubated with Renca cells inactivated by pretreatment with 50 μg/ml mitomycin C for 20 min at 37°C. Culture supernatants were obtained from the incubated cells at the indicated times. Amounts of IFN-γ in the supernatants were measured using ELISA. Bars represent mean ± SD.

We examined whether the increased antitumor activity of Ad.p35 plus Ad.p40 plus Ad.CD/5-FC (Figure 4) was associated with the induction of tumor-specific CTLs, activation of NK cells, and induction of Th1 cytokines such as IL-2 and IFN-γ. Effector cells were prepared from the splenocytes of tumor-bearing mice treated with the recombinant adenoviruses. Renca cells labeled with ^51^Cr were used as target cells. A standard 4-h ^51^chromium release assay was carried out. However, we did not detect specific lysis of the labeled Renca cells (data not shown). We performed an NK cell assay using the splenocytes as effector cells and YAC-1 cells labeled with ^51^Cr as target cells (Figure 5A). The effector cells from mice treated with Ad.p35 plus Ad.p40 plus Ad.CD/5-FC lysed the target cells about 10- and 5-fold more effectively than Ad.CD/5-FC and Ad.GFP, respectively, at a 100:1 effector-to-target cell ratio. Although Ad.p35 plus Ad.p40 treatment also increased NK cell activity compared to Ad.CD/5-FC or Ad.GFP, the cytolytic activity of splenic NK cells from mice treated with this mixture was lower than that from mice treated with Ad.p35 plus Ad.p40 plus Ad.CD/5-FC. The effector cells used in the NK cell assay were mainly splenocytes from mice in which the tumors were completely regressed or small. This may account for the increased NK cell activity in the Ad.p35 plus Ad.p40 plus Ad.CD/5-FC treatment group compared to the Ad.p35 plus Ad.p40 treatment group.

We next examined whether the virus treatments induced Th1 cytokines, which may represent innate and/or adaptive immune responses. Splenocytes from tumor-bearing mice treated with the viruses were incubated with inactivated Renca cells. The IL-2 and IFN-γ contents of the culture supernatants were measured by ELISA 24 and 48 h after incubation, respectively. We detected no IL-2 induction in splenocytes from any of the treatment groups (data not shown). However, IFN-γ production was about twice as great in splenocytes from mice treated with Ad.p35 plus Ad.p40 plus Ad.CD/5-FC as in those treated with Ad.CD/5-FC or Ad.GFP (Figure 5B, *p *< 0.0179 and < 0.0394 for Ad.p35 + Ad.p40 + Ad.CD/5-FC vs Ad.GFP and Ad.CD/5-FC, respectively). Ad.p35 plus Ad.p40 treatment also increased IFN-γ, but its effectiveness was significantly less than that of Ad.p35 plus Ad.p40 plus Ad.CD/5-FC (*p *< 0.0076). The results suggest that Ad.p35 plus Ad.p40 treatment, but not Ad.CD/5-FC, may enhance NK cell activity and stimulate IFN-γ production. Thus, the enhancement of NK cell activity may be partially responsible for the inhibition of tumor growth by treatment with Ad.p35 plus Ad.p40 plus Ad.CD/5-FC or with Ad.p35 plus Ad.p40.

**Figure 6:**
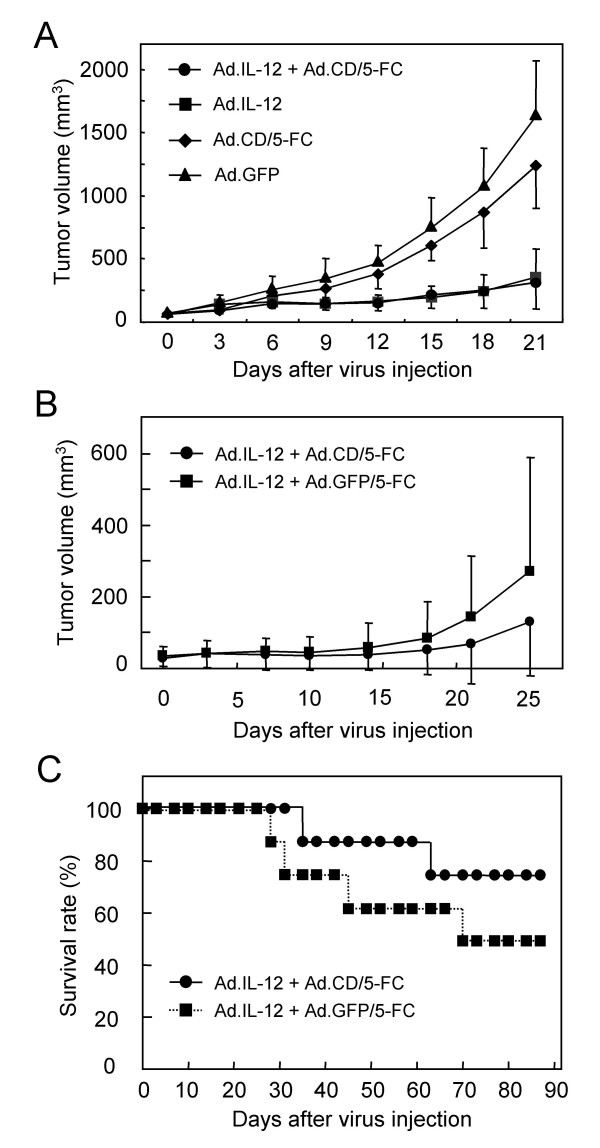
CD gene transfer followed by 5-FC treatment increases the antitumor effectiveness of IL-12 gene transfer. (A) Mice with tumors were established by subcutaneous inoculation of 1.5 × 10^5 ^cells of Renca. The experimental animals in which tumors reached a diameter of about 5 mm were randomly divided into 4 treatment groups (n = 7 per group) and injected once intratumorally (day 0) with Ad.IL-12 (5 × 10^8 ^pfu) plus Ad.CD (5 × 10^8 ^pfu), or 1 × 10^9 ^pfu of Ad.IL-12, or Ad.CD, or Ad.GFP. Mice treated with Ad.CD were given intraperitoneal 5-FC (400 mg/kg) daily for 10 days. Tumor growth was measured at intervals of three days until day 21. Graphic representations of mean tumor growth rates are shown. (B) and (C) Renca tumor-bearing mice were divided into two groups (n = 8 per group), which received single intratumorally injections (day 0) of Ad.IL-12 (5 × 10^8 ^pfu) plus Ad.CD (5 × 10^8 ^pfu) or Ad.IL-12 (5 × 10^8 ^pfu) plus Ad.GFP (5 × 10^8 ^pfu). Mice in both groups were given intraperitoneal 5-FC (400 mg/kg) daily for 10 days. Tumor growth was measured at the indicated times until day 25. Graphic representations of mean tumor growth rates are shown (B). Mouse survival until day 88 was recorded (C). The mean survival times (± SD) were 80.5 ± 20.8 days for the Ad.IL-12 plus Ad.CD/5-FC group and 67.3 ± 28.3 days for the Ad.IL-12 plus Ad.GFP/5-FC group. The difference between these mean survival times was statistically significant (*p *< 0.0368) by Student's *t *test.

The rapid tumor cell killing effect induced by suicide gene products induces a strong antitumor immune response, such as induction of tumor-specific CTLs and increased NK cell activity [54-57]. However, we detected neither a tumor-specific T cell immune response nor an enhanced NK cell activity after Ad.CD/5-FC treatment alone. It is possible that the antitumor activity of the Ad.CD constructed here was so weak that the tumor cells were not killed rapidly (Figure 4). However, this is unlikely, because Ad.CD/5-FC treatment increased the antitumor activity of Ad.IL-12 (see Figure 6B and 6C). Alternatively, the bystander effect, which kills neighboring tumor cells not expressing suicide genes, may have exerted a negative effect on the generation of tumor-specific CTL and/or active NK cells. Immune cells or antigen-presenting cells located close to the tumor cells could have taken up, and been killed by, the toxic metabolites generated by the suicide gene products. This may explain why the NK cell activity in Ad.CD-treated mice was significantly lower than in Ad.GFP-treated mice (Figure 5A, *p *< 0.001).

### Transfer of the *cytosine deaminase *gene followed by 5-FC treatment increases the antitumor effectiveness of IL-12 gene transfer in Renca-tumor bearing mice

Since a mixture of Ad.p35 and Ad.p40 was used in previous experiments (Figure 4), we examined whether Ad.IL-12, containing p35 and p40 in a single adenoviral vector (Figure 1), was more effective against the tumors when combined with Ad.CD/5-FC. Renca-tumor bearing mice were divided randomly into four groups, treated intratumorally with (1) Ad.IL-12 plus Ad.CD/5-FC, (2) Ad.IL-12, (3) Ad.CD/5-FC, or (4) Ad.GFP (control). Tumor sizes were measured at the indicated times (Figure 6A). There was a significant inhibition of tumor growth in mice treated with Ad.IL-12 plus Ad.CD/5-FC compared to Ad.GFP (*p *< 0.0456). The effectiveness of Ad.IL-12 (5 × 10^8 ^pfu) plus Ad.CD/5-FC (5 × 10^8 ^pfu) appeared to be similar to that of Ad.IL-12 (1 × 10^9 ^pfu) alone. This contrasts with the effectiveness of Ad.p35 plus Ad.p40 plus Ad.CD/5-FC as compared to Ad.p35 plus Ad.p40 (Figure 4). Presumably the in vivo gene delivery of the Ad.p35-Ad.p40 mixture was not so efficient as that of Ad.IL-12.

We further examined whether CD gene transfer followed by 5-FC treatment increased the antitumor effectiveness of IL-12 gene transfer. Sixteen Renca tumor-bearing mice were divided into 2 groups. Since a higher dose than 1 × 10^9 ^pfu of adenoviral vector appeared to be toxic to the mice, the total dose given was 1 × 10^9 ^pfu. Mice in each group received Ad.IL-12 (5 × 10^8 ^pfu) with either Ad.CD (5 × 10^8 ^pfu) or Ad.GFP (5 × 10^8 ^pfu) as a control. Both groups were treated with 5-FC for 10 days. Tumor sizes were measured at the indicated times (Figure 6B). Combined transfer of the IL-12 and CD/5-FC genes resulted in significant inhibition of tumor growth compared to IL-12 gene transfer alone (*p *< 0.0301). Six of the 8 mice treated with Ad.IL-12 plus Ad.CD/5-FC, but only 4 of the 8 mice treated with Ad.IL-12 plus Ad.GFP/5-FC, became tumor-free and survived at 88 days. The mean survival times were 80.5 ± 20.8 days for the Ad.IL-12 + Ad.CD/5-FC group and 67.3 ± 28.3 days for the Ad.IL-12 + Ad.GFP/5-FC group; the difference is statistically significant (*p *< 0.0368).

## Conclusion

Among various cancer gene therapies, immunogene therapy is a promising but challenging modality. Recent clinical trials using Ad.IL-12 have shown that intratumoral IL-12 gene transfer is a feasible and well-tolerated procedure but exerts only mild antitumor effects [24]. An increased Ad.IL-12 dose may increase the antitumor efficacy but might entail a systemic, toxic effect that would limit the use of Ad.IL-12 alone. In this study we have attempted to improve the antitumor efficacy in Renca tumor-bearing mice by using adenoviral vectors to transfer the IL-12 gene in combination with *cytosine deaminase*, followed by 5-FC treatment. The results show a marked inhibition of tumor growth and significant prolongation of survival time, suggesting that IL-12 plus CD gene transfer followed by 5-FC treatment may be an alternative modality for the human cancer treatment.

## Abbreviations

Ad: adenovirus; IL-12: interleukin-12; CD: cytosine deaminase; 5-FC: 5-fluorocytosine; DMEM: Dulbecco's Modified Eagle's Medium.

## Competing interests

The author(s) declare that they have no competing interests.

## Authors' contributions

Cho W-K and Yoo J constructed the adenoviral vectors and analyzed the functionality of the expressed gene products. Hwang K-S, Yun H-J, and Kim S performed the tumor growth studies in the animal model, and the immunological assays with murine splenocytes. Im DS drafted the manuscript.

## Pre-publication history

The pre-publication history for this paper can be accessed here:



## Figures and Tables

**Table 1:** Tumor regression following intratumoral injection of Ad.p35 plus Ad.p40, or Ad.CD/5-FC alone, or a combination of both

Treatment	Response (%)				
	
	*N*	Complete	Partial	Death or peritoneal metastasis	None
Ad.p35 + Ad.p40 + Ad.CD/5-FC	15	4 (27)	4 (27)	0 (0)	7 (46)
Ad.p35 + Ad.p40	15	1 (7)	2 (14)	‡2 (13)	10 (66)
Ad.CD/5-FC	15	1 (7)	0 (0)	†1 (7)	13 (86)
Ad.GFP	15	0 (0)	0 (0)	†1 (7)	14 (93)

*N *= number of Renca tumor-bearing mice injected once intratumorally with adenoviral vectors at the doses indicated in Figure 4 legend.

"Complete" refers to animals undergoing total and permanent regression of the injected tumor.

"Partial" refers to animals undergoing partial tumor regression followed by regrowth, or a significant delay in growth

"Death or peritoneal metastasis" refers to animals that died or had peritoneal metastasis during the period of investigation.

None refers to animals whose tumors have continued to grow at a rate comparable to controls.

†Death probably by toxicity.

‡Excluded due to peritoneal metastasis during the experiment.
